# MicroRNA-638 inhibits cell proliferation by targeting phospholipase D1 in human gastric carcinoma

**DOI:** 10.1007/s13238-015-0187-8

**Published:** 2015-08-07

**Authors:** Jiwei Zhang, Zehua Bian, Jialiang Zhou, Mingxu Song, Zhihui Liu, Yuyang Feng, Li Zhe, Binbin Zhang, Yuan Yin, Zhaohui Huang

**Affiliations:** Wuxi Oncology Institute, The Affiliated Hospital of Jiangnan University, Wuxi, 214062 China; Department of Radiation Oncology, The Affiliated Hospital of Jiangnan University, Wuxi, 214062 China; Fudan University Shanghai Cancer Center and Institutes of Biomedical Sciences, Shanghai Medical College, Fudan University, Shanghai, 200032 China

**Keywords:** gastric cancer, miR-638, cell proliferation, PLD1

## Abstract

MicroRNAs (miRNAs) are a type of small non-coding RNAs that are often play important roles in carcinogenesis, but the carcinogenic mechanism of miRNAs is still unclear. This study will investigate the function and the mechanism of miR-638 in carcinoma (GC). The expression of miR-638 in GC and the DNA copy number of miR-638 were detected by real-time PCR. The effect of miR-638 on cell proliferation was measured by counting kit-8 assay. Different assays, including bioinformatics algorithms (TargetScan and miRanda), luciferase report assay and Western blotting, were used to identify the target gene of miR-638 in GC. The expression of miR-638 target gene in clinical CRC tissues was also validated by immunohistochemical assay. From this research, we found that miR-638 was downregulated in GC tissues compared with corresponding noncancerous tissues (NCTs), and the DNA copy number of miR-638 was lower in GC than NCTs, which may induce the corresponding downregulation of miR-638 in GC. Ectopic expression of miR-638 inhibited GC cell growth *in vitro*. Subsequently, we identified that PLD1 is the target gene of miR-638 in GC, and silencing PLD1 expression phenocopied the inhibitory effect of miR-638 on GC cell proliferation. Furthermore, we observed that PLD1 was overexpressed in GC tissues, and high expression of PLD1 in GC predicted poor overall survival. In summary, we revealed that miR-638 functions as a tumor suppressor in GC through inhibiting PLD1.

**Electronic supplementary material:**

The online version of this article (doi:10.1007/s13238-015-0187-8) contains supplementary material, which is available to authorized users.

## INTRODUCTION

MicroRNAs (miRNAs) are endogenous 19–25 nt RNAs that play important gene-regulatory roles in animals and plants through binding to the 3′untranslated region (3′UTR) of target mRNAs, and they can take part in cell development, differentiation and proliferation (Bartel, 2009; Sreekumar et al., 2011). Studies have shown that miRNAs played important roles in the progression of various cancers over the past few years, including gastric carcinoma (GC) (Esquela-Kerscher and Slack, 2006; Cho, 2010). However, the biological functions and molecular mechanisms of many miRNAs in GC remain unclear.

GC is one of the most common human malignant cancers worldwide, about one million new GC cases and 723,000 deaths are estimated to have occurred in 2012, accounting for 8% of the total cases and 10% of total deaths (http://globocan.iarc.fr). Over 70% of new cases and deaths occur in developing countries (Jemal et al., 2011). Many miRNAs were reported to be associated with GC development in the past decade, including miR-146a (Yao et al., 2013), miR-204-5p (Zhang et al., 2015), miR-486 (Oh et al., 2011), miR-192 (Jin et al., 2011), miR-215 (Jin et al., 2011), miR-34b/c (Suzuki et al., 2010), miR-29a (Cui et al., 2011), miR-409-3p (Li et al., 2012a), and miR-296-5p (Li et al., 2014). These reports indicate that miRNAs play important roles in CRC development and progression, providing new avenues for GC diagnostic and therapeutic application (Ma et al., 2012). Although many research teams have developed new GC therapies recently years, further deeply investigations on molecular mechanisms underlying GC carcinogenesis are urgently needed to improve GC diagnosis and therapy.

We previously identified some differentially expressed miRNAs that could serve as prognostic and diagnostic factors for colorectal cancer (CRC) and determined that some of these deregulated miRNAs appeared to be highly related to the tumor prognosis (Huang et al., 2010; Huang et al., 2011; Song et al., 2014; Yin et al., 2014; Zhang et al., 2014; Zhang et al., 2015). Among them, we found that miR-638 is also down-regulated in GC as it in CRC. In the current study, we comprehensively investigate the biological functions and underlying molecular mechanism of miR-638 in GC carcinogenesis. The enhanced expression of miR-638 can inhibit cell proliferation *in vitro*. Furthermore, phospholipase D1 (PLD1), a putative cell apoptosis suppressor in GC, is characterized as a direct and functional target of miR-638, which provides a possible regulation pathway for PLD1 and a candidate target forthediagnostics and treatment of GC.

## RESULTS

### Expression of miR-638 is down-regulated in GC tissues

**Figure 1:**
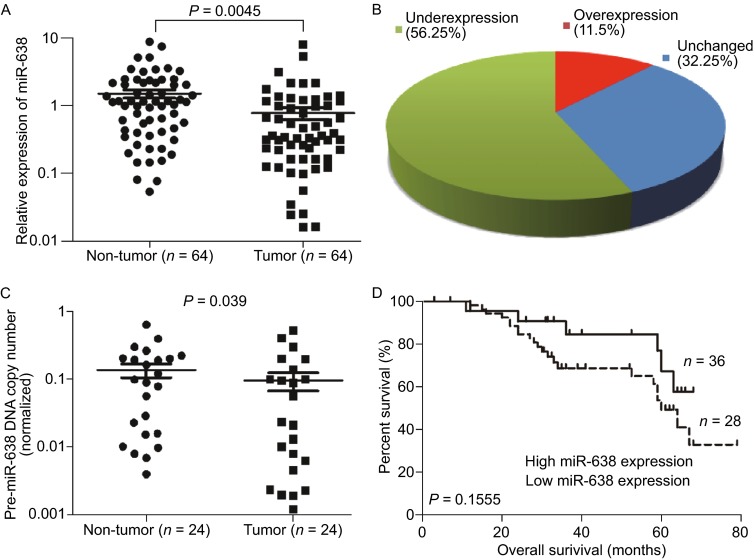
**The expression of miR-638 was down-regulated in GC**. (A) The expression of miR-638 was tested by qRT-PCR in 64 paired GC and adjacent noncancerous tissues (NCTs). (B) The levels of miR-638 were obviously down-regulated in 56.25% tumor tissues. (C) The DNA copy number of miR-638 was checked by qPCR in 24 paired GC and NCTs. (D) Kaplan-Meier analysis of the effect of the miR-638 expression on overall survival of 64 GC patients

As our previous research has found that miR-638 is down-regulated in CRC (Huang et al., 2011; Zhang et al., 2014), we intend to clarify whether miR-638 is down-regulated in GC. To validate this issue, we examined the mature miR-638 expression level in 64 GC tissues using qRT-PCR. The result showed that the miR-638 expression was down-regulated in 36 of 64 (56.25%) GC tissues when compared to the corresponding NCTs (*P* < 0.001, Fig. 1A and 1B). Because the deletion of DNA sequence coding for miRNAs can result in the downregulation of corresponding miRNAs, so we checked the pri-miR-638 copy number by qPCR in 24 pairs of GC tissues and NCTs to determine the reason of miR-638 downregulation in GC. Interestingly, we found that pri-miR-638 copy number was lower in GC tissues compared with their NCT counterparts (*P* < 0.05, Fig. 1C), suggesting that down-regulation of miR-638 in GC was modulated by the loss of DNA copy number.

However, we did not observe significant association between miR-638 expression in GC tissues and overall survival (OS) of GC patients (*P* > 0.05, Fig. 1D). In addition, miR-638 expression was not significantly associated with gender, age, tumor location, size, stage, and grading (*P* > 0.05).

### MiR-638 represses GC cell proliferation *in vitro*

**Figure 2:**
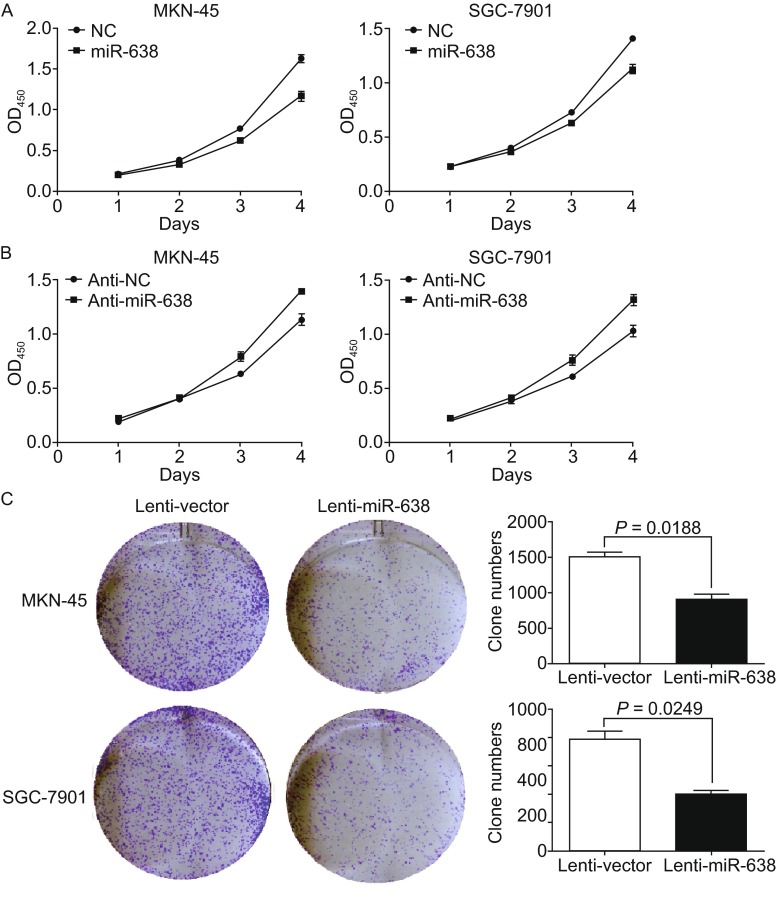
**MiR-638 inhibits GC cell proliferation** ***in vitro***. (A and B) Ectopic expression of miR-638 repressed the proliferation of MKN-45 and SGC-7901 cells, whereas silencing miR-638 expression enhanced the cellular growth rate of MKN-45 and SGC-7901 cells. (*P < 0.05). (C) The colony formation assay was also applied to confirm the ability of miR-638 on colony formation

The down-regulation of miR-638 in GC suggests that it may involve in GC tumorigenesis. Cell proliferation assay revealed that miR-638 overexpression significantly reduced the growth rates of MKN-45 and SGC-7901 cells (*P* < 0.05, Fig. 2A), and colony formation assay confirmed that miR-638 inhibited the proliferation function of GC cells (*P* < 0.05, Fig. 2C). In contrast, silencing miR-638 expression significantly promoted the growth of MKN-45 and SGC-7901 cells (*P* < 0.05, Fig. 2B). In conclusion, all these data show that miR-638 has the function of growth inhibitory.

### MiR-638 directly binds to the 3′UTR of PLD1

**Figure 3:**
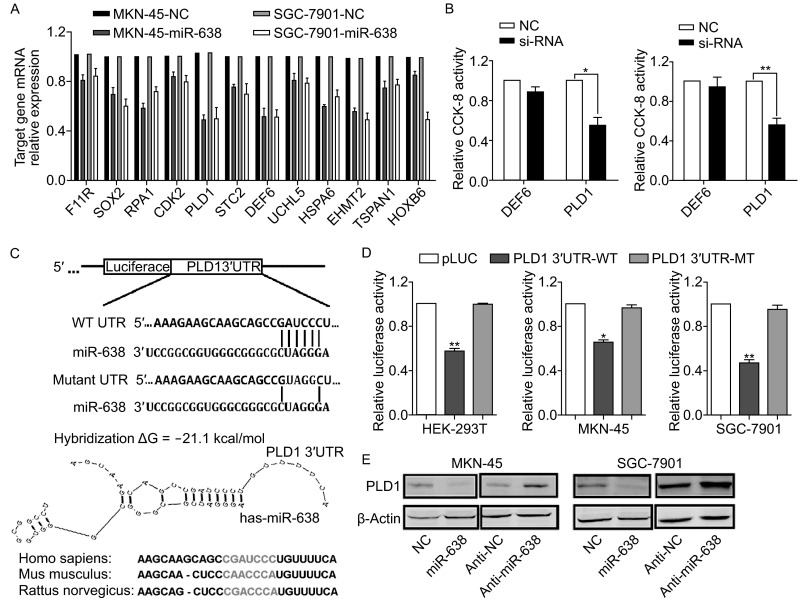
**Identification of PLD1 as the target of miR-638**. (A) Validation of the microarray results in both MKN-45 and SGC-7901 cells using qRT-PCR. A panel of 12 genes were indeed down-regulated by miR-638. (B) Proliferation assays performed on MKN-45 and SGC-7901 cells transfected with si-PLD1 or si-DEF6. Depleted PLD1 expression showed the most obvious growth repression effect. (C) Schematic of the wild-type (WT) or mutant-type (MT) 3′UTRs of the PLD1 plasmids. The complementary site of the seed region of miR-638 was selected for mutation. The free energy of hybrids between miR-638 and PLD1 was −21.1 kcal/mol. (D) Relative luciferase activity assays of luciferase reporter plasmids containing PLD1WT or MT 3′UTR were performed in cells (HEK-293T, MKN-45, and SGC-7901). Luciferase activity was determined 48 h after transfection and normalized to the Renilla luciferase activity. (E) The protein levels of PLD1 were determined by Western blotting in MKN-45 and SGC-7901 cells transfected with miR-638 mimic, miR-638 inhibitor or the corresponding NC. Beta-actin served as an internal control

To elucidate the mechanism underlying miR-638-mediated suppression of cell proliferation, we used two computer-aided algorithms, TargetScan and miRanda, to search for potential target genes of miR-638. Among the 12 potential tumor-related targets, 7 genes were significantly down-regulated in MKN-45 and SGC-7901 cells transfected with miR-638 and were considered to be the candidate targets of miR-638 (Fig. 3A). Based on these data and our previous results in CRC (Zhang et al., 2014), we focused on PLD1 and DEF6 for the subsequent analysis. The effects of the two candidate targets on cell proliferation were performed on MKN-45 and SGC-7901 cells using PLD1- or DEF6-specific siRNA. The results indicated that silencing PLD1 expression significantly decreased cell proliferation in both of MKN-45 and SGC-7901, which was not observed in the GC cells with silenced DEF6 expression (Fig. 3B). Taken together, these results suggest that PLD1 is a potential target gene of miR-638 in GC.

To further verify that PLD1 is a direct target gene of miR-638 in human GC, we generated a luciferase reporter plasmid which carried the mutated binding site of miR-638 in the PLD1 3′UTR. (Fig. 3C), and found that miR-638 had no effect on the activity of luciferase with mutant 3′UTR of PLD1 in HEK-293T, MKN-45, and SGC-7901 (Fig. 3D), suggesting the sequence-specific inhibiting of miR-638 on the PLD1 expression. In line with these results, the endogenous PLD1 protein level was also decreased in miR-638-overexpression GC cells and could be restored in miR-683-depleted GC cells (Fig. 3E).

### MiR-638 represses proliferation through directly targeting PLD1 in human GC cells

**Figure 4:**
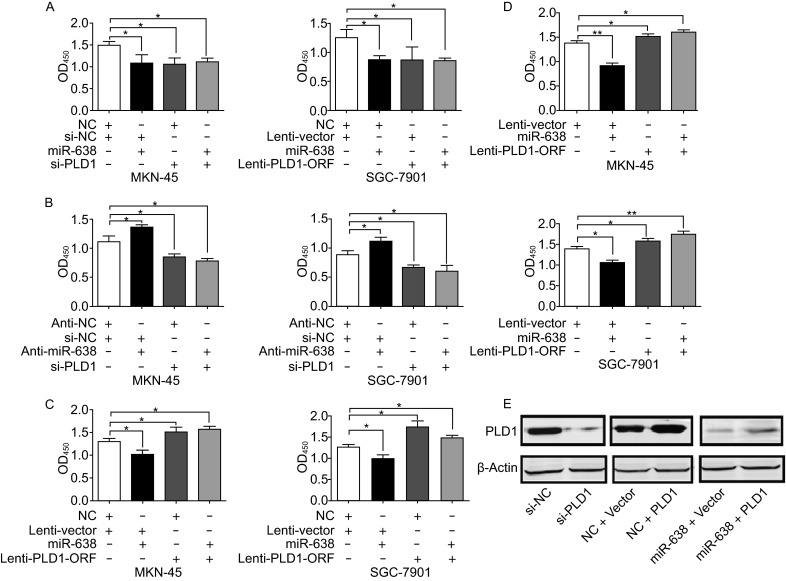
**MiR-638 repressed GC cell proliferation by inhibiting PLD1**. (A) PLD1 knockdown repressed MKN-45 and SGC-7901 cells growth, whereas upregulation of miR-638 in PLD1-depleted cells did not repress cell proliferation further. (B) MiR-638 silencing promoted cell growth, but did not promote cell proliferation in PLD1-depleted GC cells. (C) The upregulation of PLD1 ORF markedly promoted cell growth and abrogated miR-638-induced cell growth inhibition in MKN-45 and SGC-7901 cells. (D) MiR-638 overexpression promoted apoptosis, PLD1 overexpression repressed cell apoptosis and decreased the percentage of apoptotic cells after miR-638 overexpression. (E) The protein level of PLD1 was measured by Western blotting. Significant differences are indicated with * (*P < 0.05; **P < 0.01)

To further examine whether PLD1 is a direct functional target of miR-638 in GC cells, we performed a series of functional restoration assays. As shown in Fig. 4A, the proliferation of MKN-45 and SGC-7901 cells transfected with si-PLD1 were significantly decreased (Fig. 4A). Also, in miR-638 transfection group, the proliferation ability of GC cells were decreased, whereas anti-miR-638 could not restore cell proliferation in PLD1-knockdown GC cells (Fig. 4B). Furthermore, we revealed that PLD1 over-expression could significantly promoted cell proliferation, which could not be repressed by either the exogenous or endogenous overexpression of miR-638 (Fig. 4C–E). Taken together, all of these results proved that miR-638 inhibits cell proliferation via directly targeting PLD1.

### PLD1 levels are negatively correlated with miR-638 expression and GC prognosis

**Figure 5:**
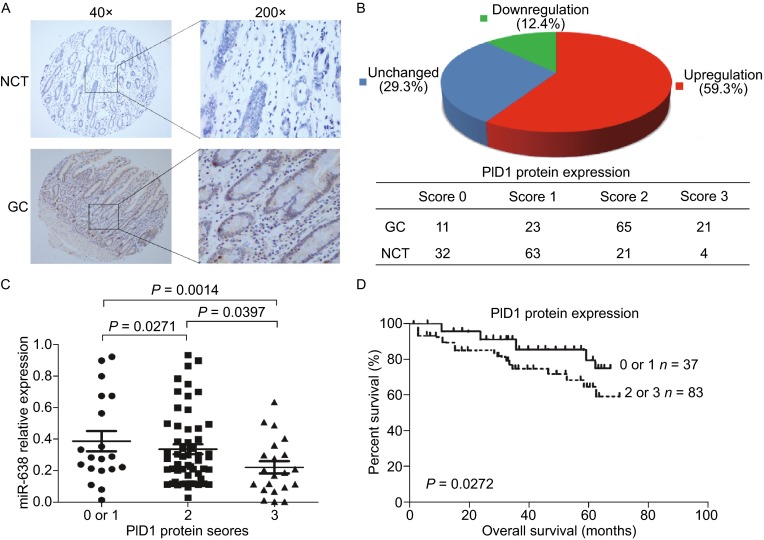
**Upregulation of PLD1 is inversely correlated with the miR-638 expression in GC**. (A) Immunohistochemical staining of PLD1 in 120 tumor tissues and adjacent noncancerous tissues (NCTs). Brown cytoplasmic PLD1 staining was observed in GC cells but was nearly absent in normal mucosal epithelia. (B) PLD1 protein expression was frequently increased in the tumor tissues (59.3%) compared with the matched NCTs. (C) The expression levels of PLD1 were negatively correlated with the miR-638 expression levels in the GC tissues (*P* = 0.0062). (D) Overall survival analysis based on the expression levels of PLD1. The groups were ranked according to the PLD1 staining intensity. The patients with high PLD1 expression (scored 2 or 3) showed poor prognosis compared with low PLD1 expression (scored 0 or 1) patients (*P* = 0.0272)

To further evaluate the relationship between miR-638 and PLD1 in human GC, PLD1 expression levels in GC tissues and paired NCTs were examined by IHC assay. As Fig. 5A and 5B showed, PLD1 protein expression in GC was upregulated in 71/120 cases compared with paired NCTs, and was inversely correlated with the miR-638 levels (*P* < 0.05, Fig. 5C), which suggested that the increased PLD1 expression in GC was caused by miR-638 under-expression. Survival analyses revealed that increased PLD1 protein levels (score 2 or 3) were associated with shorter survival time (*P* = 0.0272, Fig. 5D). After adjusting from tumor size, grading, and stage, multivariate analyses showed that PLD1 expression was an independent risk factor for survival (Table S1). Patients with higher PLD1 expression presented a higher risk of death (HR = 3.3049, 95% CI = 1.213–6.375, *P* < 0.05) (Tables S1 and S2). Taken together, these data suggest that PLD1 appears to be a new prognostic factor for GC.

## DISCUSSION

Nowadays, more than 2,000 miRNAs have been identified in human, as an important gene regulated factor, it has been identified as a key regulation factor for the tumor cell proliferation, migration, and invasion. Down-regulation of miR-638 was first identified in human non-small-cell lung cancer (NSCLC) tissues (Li et al., 2012b). Our previous work showed that miR-638 was downregulated in human CRC, and loss of miR-638 promotes CRC cell proliferation (Zhang et al., 2014). Other groups found that the loss of miR-638 could promote cell invasion and a mesenchymal-like transition in CRC cells (Ma et al., 2014), and downregulation of miR-638 promotes cell invasion and proliferation in NSCLS (Xia et al., 2014). These data suggest a key role of miR-638 in human tumorigenesis.

GC is the second leading cause of global cancer mortality and most of GC patients are diagnosed with advanced-stage diseases (Cidon et al., 2013). As miR-638 had been verified to be down-regulated in CRC, we want to clarify whether it is also abnormal in human GC. We checked the expression of miR-638 in 64 paired of GC tissues and NCTs, and observed significantly down-regulation of miR-638 in GC, which was in consistent with a previous report (Shrestha et al., 2014).

The miR-638 gene is an intronic miRNA located on the first intron of DNM2. Our previous study revealed promoter methylation is a potential mechanism accounting for the down-regulation of the miR-638 in CRC. Copy number variants (CNV) are the other notable structural variations in the human genome that influence gene expression. Several studies have examined the CNVs of miRNAs and revealed their potential consequences (Ding et al., 2010; Wang et al., 2014). We observed that the copy numbers of pri-miR-638 were decreased in GC compared with NCT samples, suggesting that it account for, at least partly, the downregulation of miR-638 in GC. However, for the relative small case number in this study, these findings should be confirmed in an enlarged GC cohort in the future study.

In this study, we found that loss of miR-638 repressed cell proliferation and colony formation in GC via its direct target, PLD1. In addition to affecting proliferation, the role of miR-638 in metastasis is another important aspect that must be considered in future researches. PLD1 is an enzyme that can convert phospholipid to phosphatidic acid (PA), and two isoforms of phosphatidylcholine-specific PLD, PLD1 and PLD2 have been identified. PLD is upregulated at protein and/or activity levels in various cancers (Zhang and Frohman, 2014). Our present study showed that PLD1 is over-expressed in GC tissues compared with adjacent NCTs, and PLD1 appears to be a new prognostic factor for GC. Although upregulation of PLD1 plays an important role in increasing cell proliferation, the molecular mechanisms mediating PLD1 expression in GC cells are still unclear. The expression of PLD1 was reported to be induced in GC cells by infecting with cagA-positive H. pylori (Kang et al., 2013). We reported here that loss of miR-638 caused overexpression of PLD1 in GC cells. This mechanism explicated the reason of PLD1 overexpression in GC cells in a post-transcriptional regulation level. The present study showed that miR-638 is frequently down-regulated in GC, which partly attributed to the copy number of miR-638 locus; and miR-638 functions as a new repressor of GC cell proliferation. In addition, PLD1 identified as functional target of miR-638 in this study, was upregulated in GC and was closely related to the prognosis of GC patients. These results suggest that miR-638/PLD1 signalling could be a useful marker in GC and potential therapeutic targets for the treatment of GC patients.

## MATERIALS AND METHODS

### Cell lines and clinical samples

HEK-293T cell line was purchased from the American Type Culture Collection (ATCC), and human GC cell lines (MKN-45 and SGC-7901) were purchased from Shanghai Meixuan. All of the media (Hyclone, USA) were supplemented with 10% fetal bovine serum (FBS) (Gibco, USA). SGC-7901, MKN45, and HEK-293T cells were cultured in DMEM.

A total of 64 pairs of human primary GC tissues and their adjacent NCTs were collected between 2008 and 2012 at the Affiliated Hospital of Jiangnan University. The tissue samples were immediately snap-frozen in liquid nitrogen and were histologically confirmed. All of the human materials were obtained with informed consent, and this project was approved by the Clinical Research Ethics Committee of Affiliated Hospital of Jiangnan University.

### DNA and RNA isolation

Genomic DNA was isolated using the General AllgGen Kit (Cwbio, China) according to the manufacturer’s protocol. Total RNA was extracted using RNAiso reagent (Takara, Japan). The concentrations of DNA and RNA were determined using NanoDrop 2000 (Thermo, USA).

Complementary DNA (cDNA) was synthesized using the PrimeScript RT reagent kit (TaKaRa, Japan). QRT-PCR analyses were conducted to quantitate the relative mRNA expression using SYBR Premix Ex Taq (TaKaRa), with β-actin as an internal control. Stem-loop qRT-PCR assays using TaqMan miRNA probes (Applied Biosystems, USA) were performed to quantify the levels of the mature miRNAs. The reactions were incubated in 96- or 384-well optical plates at 95°C for 10 min, followed by 40 cycles of 95°C for 15 s and 60°C for 1 min. After the reactions, the cycle threshold (Ct) data were determined using default threshold settings, and the mean Ct was determined from the duplicate PCRs. A comparative ΔCt method was used to compare each condition with the controls, and the values are expressed as 2^−△Ct^. The relative levels of miRNAs were normalized to the levels of U6, a ubiquitously expressed small nuclear RNA. The relative DNA copy numbers were determined as described previously (Wang et al., 2002).

### Plasmid and siRNA

The miR-638 lentivirus expression vector pWPXL-miR-638 was constructed as described (Zhang et al., 2014). The 3′UTRs of potential miR-638 target genes were amplified from genomic DNA using PrimerSTAR Premix (TaKaRa). The amplified 3′UTRs were then cloned into the region directly downstream of a CMV promoter-driven firefly luciferase cassette in a pcDNA3.0 vector (p-Luc). The mutant 3′-UTR of PLD1, which carried the mutated sequence in the complementary site for the seed region of miR-638, was constructed based on the p-Luc-PLD1 3′UTR-WT plasmid by overlap-extension PCR. The open reading frame (ORF) of PLD1 was amplified and cloned into pWPXL. Duplex siRNAs were purchased from GenePharma (Shanghai, China). The anti-miRNA inhibitors (GenePharma, China) are single-stranded nucleic acids chemically modified to specifically bind to and inhibit endogenous miRNA molecules.

### Lentivirus production and transduction

Virus packaging was performed in HEK 293T cells after co-transfection of pWPXL-638 or pWPXL-PLD1 with the packaging plasmid psPAX2 and envelope plasmid pMD2.G using Lipofectamine 2000 (Invitrogen). Viruses were harvested 48 h after transfection, and viral titers were determined. Target cells (1 × 10^5^), including MKN-45 and SGC-7901 cells, were infected with 1 × 10^6^ recombinant lentivirus-transducing units in the presence of 6 μg/mL polybrene (Sigma, MO).

### Cell proliferation assay and colony formation assay

As the cell proliferation was quantified using the Cell Counting Kit-8 (CCK8; Dojindo Laboratories, Japan) according to the manufacturer’s instructions. For the colony formation assays, 1000 cells of MKN-45 and 1500 cells of SGC-7901 cells were plated into each well of 6-well plates and incubated in medium containing 10% FBS for 2 weeks. The colonies were fixed with methanol and stained with 0.1% crystal violet in 20% methanol for 20 min. The number of colonies containing more than about 30 cells was counted using an inverted microscope.

### Luciferase assay

Approximately 5,000 HEK-293T cells or 10,000 GC cells (MKN-45 and SGC-7901) per well were plated into 96-well plates and were cotransfected with 50 nmol/L of miR-638 mimic (or NC), 50 ng of the luciferase reporter, and 10 ng of the pRL-CMV Renilla luciferase reporter using 0.5 μL Lipofectamine 2000 (Invitrogen, USA) per well. After 48-h of transfection, the luciferase activities were quantified using a dual-luciferase reporter assay (Promega, USA).

### Western blot

Harvested proteins were first separated by 10% sodium dodecyl sulphate-polyacrylamide gel electrophoresis and then transferred to nitrocellulose membranes (Millipore, USA). The membranes were blocked with 5% nonfat milk and incubated with a mouse anti-PLD1 polyclonal antibody at a dilution of 1:500 (Cell signal, USA) or a mouse anti-beta-actin monoclonal antibody at a dilution of 1:500 (Sigma, USA). The membranes were subsequently incubated with a goat anti-mouse horseradish peroxidase secondary antibody (Sigma, USA). The protein complex was detected using enhanced chemiluminescence reagents (Pierce, France). Endogenous beta-actin was used as the internal control.

### Immunohistochemistry (IHC)

Tissue arrays were constructed using 120 paired GC tissues and NCTs. Immunohistochemical staining was performed on 4 μm sections of paraffin-embedded tissues to determine the expression level of PLD1 protein. In brief, the slides were incubated in PLD1 antibody (Cell signal, USA) diluted 1:400 at 4°C overnight. The subsequent steps were performed using the EnVision™ FLEX High pH visualisation system according to the manufacturer’s instructions (DAKO, Demark). The PLD1 protein score was estimated based on the overall staining strength.

### Statistical analysis

The results were presented as the mean ± standard error of the mean (SEM). The data were subjected to two-tailed Student *t* test (*P* < 0.05) unless otherwise specified (χ2 test or Spearman’s correlation). Cox proportional hazards regression analysis was used to estimate the hazard ratios (HRs) and the 95% confidence intervals (CIs). A value of *P* < 0.05 was considered to be statistically significant. The SPSS 16.0 package (IBM, USA) was used for the statistical analyses.

## Electronic supplementary material

Supplementary material 1 (DOCX 25 kb)
