# Supplementary material for: MicroRNA-638 inhibits cell proliferation by targeting phospholipase D1 in human gastric carcinoma
Source: Protein Cell. 2015 Aug 7;6(9):680–8. doi: 10.1007/s13238-015-0187-8 (PMC4537476; doi:10.1007/s13238-015-0187-8)
Supplement: Supplementary file 1 — Supplementary material 1 (DOCX 25 kb) [file 13238_2015_187_MOESM1_ESM.docx]

**Support table**

**Table 1. Relationship between PLD1 expression and clinicopathologic factors of patients with Gastric carcinoma.**

| Parameter | No. of patients | PLD1(low) | PLD1 (high) | *P* -value |
| --- | --- | --- | --- | --- |
| Sex |  |  |  | 0.5604 |
| male | 64 | 29 | 35 |  |
| female | 56 | 22 | 34 |  |
| Age (yr) |  |  |  | 0.2041 |
| ＜ 60 | 69 | 32 | 37 |  |
| ≥ 60 | 51 | 20 | 31 |  |
| Tumor differentiation |  |  |  | **0.0054** |
| Ⅰ | 6 | 4 | 2 |  |
| Ⅱ | 65 | 22 | 43 |  |
| Ⅲ | 49 | 18 | 31 |  |
| Tumor size (cm) |  |  |  | **0.0211** |
| ≤5 | 42 | 19 | 23 |  |
| ＞5 | 78 | 26 | 52 |  |
| Differentiation grade |  |  |  | 0.0891 |
| Well-moderate | 58 | 23 | 35 |  |
| Poor-undifferentiation | 62 | 30 | 32 |  |
| T stage |  |  |  | **0.0042** |
| T1-T3 | 41 | 19 | 22 |  |
| T4 | 79 | 15 | 64 |  |
| Lymph node status |  |  |  |  |
| Negative | 57 | 30 | 27 | 0.412 |
| Positive | 63 | 28 | 35 |  |
| Distant metastasis |  |  |  | 0.2137 |
| M0 | 59 | 34 | 25 |  |
| M1 | 61 | 24 | 37 |  |
| TNM stage |  |  |  | 0.0654 |
| I-II | 56 | 26 | 30 |  |
| III-IV | 64 | 21 | 43 |  |
| Lymphatic invasion |  |  |  | 0.0621 |
| Negative | 51 | 26 | 25 |  |
| Positive | 69 | 24 | 45 |  |
| Venous invasion |  |  |  | 0.0511 |
| Negative | 68 | 31 | 37 |  |
| Positive | 52 | 27 | 25 |  |

**Table 2. Univariate analysis identifies factors influencing the overall survival rate of Gastric carcinoma patients.**

| Factors | HR | 95% CT | P value |
| --- | --- | --- | --- |
| Sex | 0.997 | 1.01-2.35 | 0.481 |
| Age（>60 vs.≤60） | 1.365 | 0.902-2.624 | 0.523 |
| Tumor size(cm)>5 | 5.627 | 5.014-17.65 | **0.0234** |
| Tumor size(cm)<5 | 3.124 | 0.734-4.012 | 0.0602 |
| Differentiation grade | 2.221 | 1.356-3.621 | **0.0341** |
| T stage | 1.89 | 1.237-2.702 | **0.0045** |
| Lymph node status | 2.967 | 0.98-3.02 | **0.013** |
| Distant metastasis | 2.24 | 0.896-2.354 | 0.367 |
| TNM stage（Ⅲ vs.Ⅱvs.Ⅰ） | 1.4.5 | 1.001-3.254 | **0.0211** |
| Lymphatic invasion | 1.67 | 1.07-3.024 | **0.0071** |
| Venous invasion | 1.79 | 1.09-3.32 | 0.436 |
| PLD1 expression | 2.57 | 1.96-2.986 | **0.0024** |

**HR: hazard ratio; CI: confidence interval; TNM: tumor–node–metastasis classifications.**

**Table 3. Multivariate analysis identifies factors influencing the overall survival rate of Gastric carcinoma patients.**

| Factors | HR | 95% CT | P value |
| --- | --- | --- | --- |
| Tumor size(cm)>5 vs. ≤5 | 1.97 | 1.21-2.64 | **0.027** |
| Differentiation | 1.79 | 1.01-2.45 | 0.320 |
| T stage | 0.97 | 0.78-2.14 | **0.001** |
| Lymph node status | 2.63 | 1.14-2.34 | 0.053 |
| TNM stage(Ⅲvs.Ⅱvs.Ⅰ） | 1.25 | 1.13-1.97 | **0.011** |
| Lymphatic invasion | 0.97 | 1.02-3.13 | 0.081 |
| Venous invasion | 1.103 | 2.01-3.436 | 0.425 |
| PLD1 expression | 2.33 | 1.15-3.26 | **0.002** |

HR: hazard ratio; CI: confidence interval; TNM: tumor–node–metastasis classifications.
